# Effect of kaempferol ingestion on physical activity and sleep quality: a double-blind, placebo-controlled, randomized, crossover trial

**DOI:** 10.3389/fnut.2024.1386389

**Published:** 2024-08-02

**Authors:** Yasutaka Ikeda, Aina Gotoh-Katoh, Shinpei Okada, Shuichi Handa, Teruyuki Sato, Tsubasa Mizokami, Bungo Saito

**Affiliations:** ^1^Otsu Nutraceuticals Research Institute, Otsuka Pharmaceutical Co. Ltd., Otsu, Japan; ^2^Physical Education and Medicine Research Foundation, Tomi, Japan; ^3^Saga Nutraceuticals Research Institute, Otsuka Pharmaceutical Co. Ltd., Saga, Japan; ^4^Tomi City Mimaki Onsen Clinic, Tomi, Japan

**Keywords:** behavioral change, daily step count, sleep quality, Fitbit, kaempferol

## Abstract

**Background:**

Kaempferol (KMP), a flavonoid in edible plants, exhibits diverse pharmacological effects. Growing body of evidence associates extended lifespan with physical activity (PA) and sleep, but KMP’s impact on these behaviors is unclear. This double-blind, placebo-controlled, crossover trial assessed KMP’s effects on PA and sleep.

**Methods:**

A total of 33 city workers (17 males and 16 females) participated in this study. They were randomly assigned to take either 10 mg of KMP or placebo for 2 weeks in the order allocated, with a 7-day washout period in between. All participants wore an accelerometer-based wearable device (Fitbit Charge 4), which monitored daily PA, heart rate (HR), and HR variability during sleep.

**Results:**

The duration of wearing the device was 23.73 ± 0.04 h/day. HR decreased in each PA level, and the mean daily step count and distance covered increased significantly during KMP intake compared to placebo. The outing rate, number of trips, number of recreational activities, and time spent in recreation on weekends increased. Sleep quality improved following KMP intake. The decrease in HR and increase in RMSSD may be important in mediating the effects of these KMPs.

**Conclusion:**

KMP leads to behavioral changes that subsequently improve sleep quality and potentially improve long-term quality of life.

**Clinical Trial Registration:**

https://center6.umin.ac.jp/cgi-open-bin/ctr_e/ctr_view.cgi?recptno=R000048447, UMIN000042438.

## 1 Introduction

Advancements in medicine, technology, and dietary preferences have ushered in an era where people can potentially live up to 100 years (1). The compelling health benefits of physical activity (PA) and quality sleep are undeniable, with increased daily PA substantially lowering the risk of premature death and mitigating over 25 chronic health conditions (2, 3). The potential of physical inactivity to heighten the risk of various diseases and increase the all-cause mortality rate is widely recognized. Therefore, it is universally acknowledged that moderate exercise is vital for maintaining health and preventing diseases (4–6). An increase of merely 1,500 steps in average daily step count corresponds to an impressive 2.2% reduction in mortality risk (7). Reduced PA is the fourth leading risk factor for premature death, behind hypertension, smoking, and diabetes mellitus (8). Despite the numerous initiatives aimed at promoting PA, there has been little progress in improving these outcomes (9).

PA strongly correlates with maximal oxygen uptake (VO_2max_) (10, 11), a key metric of aerobic capacity. It plays a fundamental role in mitigating disease risk through diverse mechanisms, including weight loss, inflammation reduction, and promoting mental well-being (12). As individuals age, the decline in mitochondrial function, which is closely associated with PA, leads to a decrease in VO_2max_, consequently, resulting in reduced daytime activity and compromised sleep quality (13, 14). Strategies to enhance or maintain PA levels typically involve raising awareness through social initiatives, formulating guidelines, and using educational methods. Nutritional approaches are being explored to improve sleep quality, and specific food components such as tryptophan, melatonin, serotonin, and glycine directly enhance sleep quality (15, 16). Saturated fatty acids and proteins can affect sleep quality through the secretion and synthesis of serotonin (15). However, if the decrease in daytime PA is primarily due to mitochondrial dysfunction, which leads to reduced oxygen uptake and energy supply, these strategies might not be effective. In such scenarios, increasing daytime PA emerges as a promising solution. Previous studies predominantly focused on PA’s effects on sleep in adults with specific illnesses, health issues, or clinical sleep disorders (17), leaving a gap in understanding how daily PA affects sleep in healthy adults. In recent years, direct-to-consumer wearable devices have become valuable in behavioral research. In particular, the Fitbit activity trackers (Fitbit, San Francisco, California, United States) are highly regarded for their superior accuracy among commercially available wearable devices (18–23), capable of tracking PA, heart rate (HR), global positioning system (GPS) location, and sleep. In particular, this tracker is regarded as a useful device for measuring PA (18) and step counts (22, 23), making it suitable for this trial. This technology, which allows for the continuous monitoring of one’s overall daily life and health status, holds promise for scientific research and enables individuals to better understand themselves and naturally heighten their health consciousness.

Kaempferol (KMP), a flavonoid in various edible plants, is renowned for its antioxidant, anti-inflammatory, anticancer, cardioprotective, and neuroprotective properties (24–26). In the *in vitro* assay using C_2_C_12_ cells, we found that KMP activates mitochondrial oxidative metabolism and elevates intracellular adenosine triphosphate (ATP) levels under hypoxic conditions by effectively suppressing the stabilization of hypoxia-inducible factor-1α (HIF-1α) (27). It is conceivable that the function of KMP affects specific pathological conditions and broadly influences daily life; however, this aspect has not been investigated. For example, during exercise, the balance between oxygen supply and demand is disrupted in skeletal muscles, resulting in temporary hypoxia and a decrease in mitochondrial function, which reduces exercise capacity (28). HR tends to increase proportionally with accumulating fatigue during exercise (29–31), and improving VO_2max_ can improve sleep quality (14, 32). In this study, we focused on the effect of KMP based on oxygen uptake, mitochondrial function, and energy production. We utilized the abilities of the Fitbit Charge 4 device to investigate how KMP intake influences daily PA levels and sleep quality and its potential to enhance the overall quality of life. Our findings can yield societal benefits in the future. This is the first study investigating the effects of flavonoid ingestion on PA and sleep using a wearable device for comprehensive monitoring and analyses.

## 2 Materials and methods

### 2.1 Trial design and participants

Ethical considerations were strictly adhered to during this study, following the principles outlined in the Declaration of Helsinki. All procedures involving human subjects were approved by the Ethics Committee of Otsuka Pharmaceutical Co., Ltd. (approval no. 2003, dated October 30, 2020).

This double-blind, placebo-controlled, randomized, crossover study, was conducted at Saga Nutraceuticals Research Institute, Otsuka Pharmaceutical Co., Ltd. between November 2020 and December 2020. Thirty-seven untrained city workers recruited through advertisements using flyers and over email participated in the study. Written informed consent for participation and publication of the data was obtained from each participant after providing a comprehensive explanation of the experimental procedures and associated risks.

Participants visited the laboratory before the supplementation period in the two-week selection period. During this visit, their medical histories were recorded, and they underwent physical examinations, biochemical blood tests, and hematological tests, allowing physicians to assess potential health risks and chronic diseases. Participants consumed 10 mg KMP supplement and a 24 h urine collection was performed to evaluate the urinary excretion of KMP.

Based on these results, we excluded participants with digestive, circulatory, or endocrine diseases, those using medication for treatment, those having food allergies, pregnant women, or those deemed ineligible for this study by a medical doctor. Thirty-four participants proceeded to the double-blind supplementation phase. The random assignment was performed by a person blinded to this study’s conduct and analysis. Random allocation to the two groups, placebo start and KMP start, was performed using the permuted block method (block size of four) by entering the described variables (age, sex, and body mass index) into the SAS software (version 9.4; SAS Institute, Cary, NC, United States) in a 1:1 ratio. This facilitated an automatic allocation and generated a unique ID code for each participant. The personnel administering test foods to the participants differed from those performing experiments and analyzing data, ensuring blinding of the staff involved in analyses to the test foods administered to the participants.

**Figure 1:**
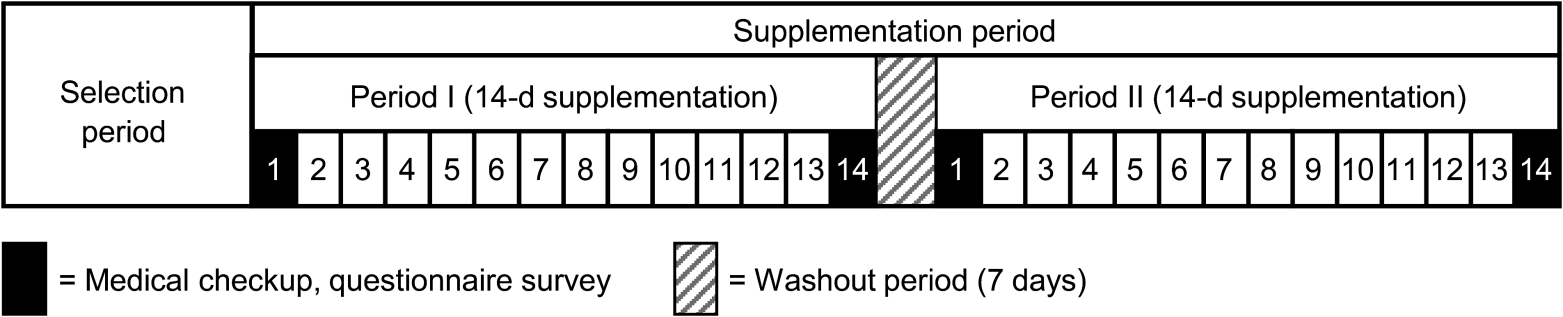
Schematic illustration of the crossover design of the study.

During the supplementation period, participants consumed either a granulated formulation containing 10 mg of KMP or a placebo granulate, designed as isocaloric, every day after breakfast for 2 weeks, with a 7-day washout period in between. Throughout this period, various parameters, such as PA levels, heart rate, and sleep indices were monitored using the Fitbit Charge 4 device. Participants maintained a diary detailing their physical condition, PA, and specifics of supplement intake. A schematic of the study design is shown in Figure 1. Participants were instructed to avoid making significant changes to their lifestyle habits during the study period or disclose any use of nutritional supplements or medication. They were directed to avoid consuming foods and beverages high in KMP content throughout the selection period.

### 2.2 Data collection using Fitbit

Each participant received a Fitbit Charge 4 tracker to wear on their non-dominant hand for the study. Detailed instructions on proper device use and mobile application installation according to the manufacturer’s specifications were provided. Additionally, participants were trained on the periodic synchronization of their Fitbit tracker to ensure accurate data recording and monitoring throughout the study.

Parameters related to PA (step count, walking and running distance, metabolic equivalent of task [MET], GPS position) and HR measurements were directly downloaded from the web server using a developer’s application programming interface (API) provided by Fitbit. This API facilitated the direct extraction of data from the Fitbit web server. The non-wear time was defined as 120 or more consecutive minutes with zero steps counted. All other time intervals were categorized as wear time (33). Minutes with over 200 steps were flagged as missing data points (34, 35). Fitbit data were considered adequate when there were at least 10 h of Fitbit wear time during a given day. This strategy was applied to guarantee the consistency and accuracy of the data collected throughout the study.

The PA levels were categorized as rest (<1.5 METs), light (1.5–2.9 METs), moderate (3–5.9 METs), and vigorous (>6 METs) from the data obtained from Fitbit. PA level and HR at each intensity were computed, and HR-MET curves were generated for each supplement intake using Microsoft Excel (Microsoft Corporation, Redmond, WA, United States). The minute-by-minute GPS data downloaded from the Fitbit application was imported into QGIS (QGIS Geographic Information System. Open Source Geospatial Foundation Project. http://qgis.org). Walking speed was calculated using the distance traveled derived from GPS coordinates during walking and was expressed as distance traveled per minute. A 5-m square grid was created around the Tomi city office and Tomi welfare center where the participants worked, and heat maps were generated based on the number of steps taken per minute. Fitbit data provided sleep information in 30-s intervals based on movement and HR data, including sleep score and stage data (sleep time, wake, light sleep, deep sleep, and rapid eye movement (REM) sleep). The HR data during sleep was used to extract the respiratory rate at 5-s intervals via the API. The root mean square of the successive differences (RMSSD) and low frequency/high frequency (LF/HF) ratio during sleep were calculated using a PC program (HRVanalysis, Université Jean Monnet Saint-Etienne, France).

### 2.3 Time-use and activity diary

The participants recorded their in-home and out-of-home daily activities (such as shopping, recreation, exercise, jog-related activities, housework, rest, and sleep) in a logbook. The accuracy of the information recorded in the logbook was cross-referenced with the GPS coordinates of the activity locations at the end of the study. The percentage of participants making at least one trip per day (outing rate), number of trips, number of recreational activities, and time spent on recreation on weekends were calculated.

### 2.4 KMP analysis in urine

Urine analysis involved mixing 100 μL of each sample with 100 μL (50 units) of β-glucuronidase solution (Sigma Aldrich, St. Louis, MO, United States) in 0.2 M sodium acetate buffer (Wako Pure Chemicals Industries Ltd., Tokyo, Japan), pH 5.0. The mixture was incubated at 37°C for 30 min, followed by the addition of 200 μL of 4% phosphate buffer containing internal standard, apigenin-d5 (Toronto Research Chemicals, Inc., North York, ON, Canada).

The prepared urine samples were transferred to a conditioned 96-well Oasis MCX μElution plate (Waters Corporation, Milford, MA, United States), passed through the sorbent bed, and washed with 200 μL of 2% formic acid (Wako Pure Chemicals Industries Ltd., Tokyo, Japan) in water, followed by 200 μL of 40% methanol (Fisher Scientific, Hampton, NH, United States). KMP and the internal standard were eluted with 150 μL methanol/acetonitrile (Fisher Scientific, Hampton, NH, United States) in another 96-well elution plate for final analysis. Chromatographic separation involved injecting 20 μL of the prepared sample into a reverse-phased C18 analytical column (50 mm × 2 mm, 3 μm particle size, Cadenza CD-C18, Imtakt Co., Kyoto, Japan). We achieved high-performance liquid chromatographic (HPLC) separation on a Shiseido Nanospace HPLC system (Tokyo, Japan). The mobile phase comprised 0.1% formic acid with water and acetonitrile, and operated at a flow rate of 0.35 mL/min. The chromatographic conditions were held constant at the initial mobile phase composition (20% acetonitrile) for 0.5 min, followed by a linear gradient to 95% acetonitrile for 2 min, maintained at 95% acetonitrile for 3.5 min, followed by a linear gradient to 20% acetonitrile for 3.6 min, and maintained a 20% acetonitrile for 5.5 min. The HPLC-separated samples were analyzed using a Sciex API-3000 tandem mass spectrometer (Sciex, Framingham, MA, United States) equipped with a Turbo Ion Spray interface.

### 2.5 Statistical analyses, PCA, and correlational analysis

All data are presented as the mean ± standard error of the mean. All statistical analyses were performed using SAS software (version 9.4), R (version 4.3.3) with packages “beeswarm” and “corrplot,” and Python (version 3.10). A *p*-value < 0.05 was considered statistically significant. The data were analyzed using a linear mixed-effects model. Sequence, period, and treatment were the fixed factors, while subjects within the period and within the subject were the random factors. We did not input the missing data into the primary model considering the mixed model of repeated measures approach.

PCA (principal component analysis) was performed using standardized data on 18 indicators, including daily step count; moving distance; total METs; total-, interrupted-, rem-. light-, and deep-sleep times; sleep score; daily, rest, light, middle, and vigorous activities; sleep-HR; RMSSD; and LF/HF. The missing data were complemented by the values calculated by applying default parameters with the KNNImputer of sci-kit-learn (v1.2.2). The differences in principal component scores (PC1 and PC2) between placebo and KMP were calculated, and the cosine similarity with the principal component loadings of each variable was calculated.

Spearman correlation analysis for effects due to KMP intake was performed between the change in the 18 indicators (Δ = KMP – Placebo) and KMP absorbability.

Spearman correlation analysis for each indicator was performed using data for the 19 parameters, including 18 indicators and KMP absorbability for placebo and KMP groups. KMP absorbability in the placebo group was set to 0.

## 3 Results

### 3.1 Participants’ information

**Figure 2:**
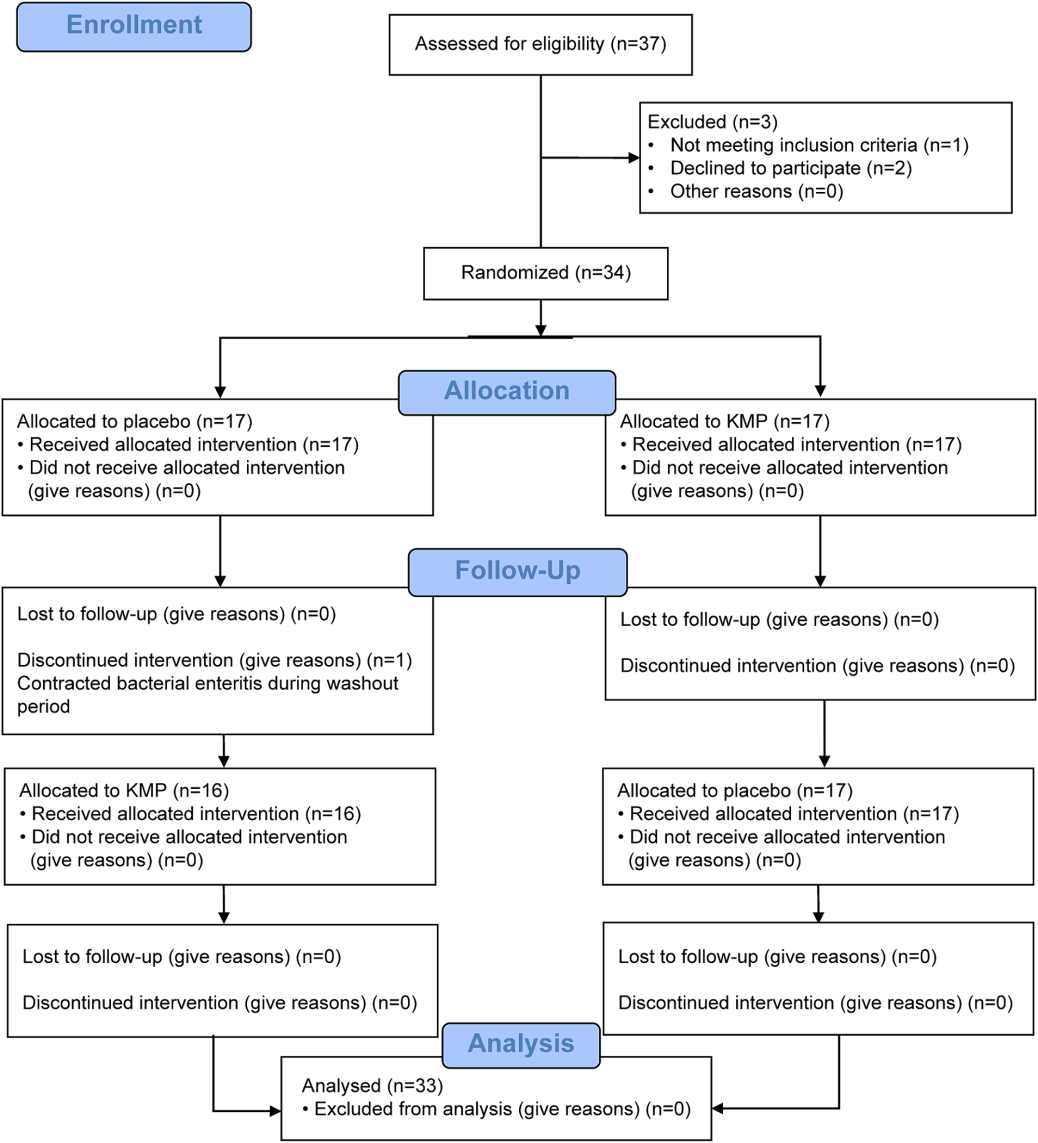
Flowchart for participant enrolment in the study. PPS, per-protocol set.

This trial comprised a selection period, 14-day food intake period, 7-day washout period, and 14-day food intake period (Figure 1). Figure 2 illustrates the CONSORT flow diagram describing the participant allocation, follow-up, and analysis process. The CONSORT checklist is provided as a Supplementary material. Participant eligibility was assessed during the recruitment period. Initially, 37 participants were screened; however, two withdrew their consent to participate and one was excluded because of medication. Thus, 34 participants were enrolled and randomly assigned to two groups. Of these, 33 completed all trial procedures. One participant suffered from bacterial enteritis during the washout phase and discontinued participation in the study.

The mean wearing time of the device was 23.73 ± 0.04 h/day. One participant did not intake the supplement for 1 day, while the remaining participants had a 100% intake rate. Per-protocol analysis (PPS) was the primary analysis of efficacy. Table 1 shows the background information of the participants whose data were included in the PPS analysis.

**Table 1:** Participants’ characteristics.

Characteristic	*n* = 33
Sex	Male, 17; Female, 16
Age (years)	42.1 ± 1.9
Height (cm)	164.6 ± 1.6
Weight (kg)	64.5 ± 2.1
Body mass index (kg/m^2^)	23.7 ± 0.6
KMP excretion (mg)	1.02 ± 0.12
Wearing time of the Fitbit (h/day)	23.73 ± 0.04

Data are presented as the mean ± standard error of the mean.

### 3.2 KMP intake resulted in a decrease in HR across all activity levels, leading to an increase in step count and moving distance

**Figure 3:**
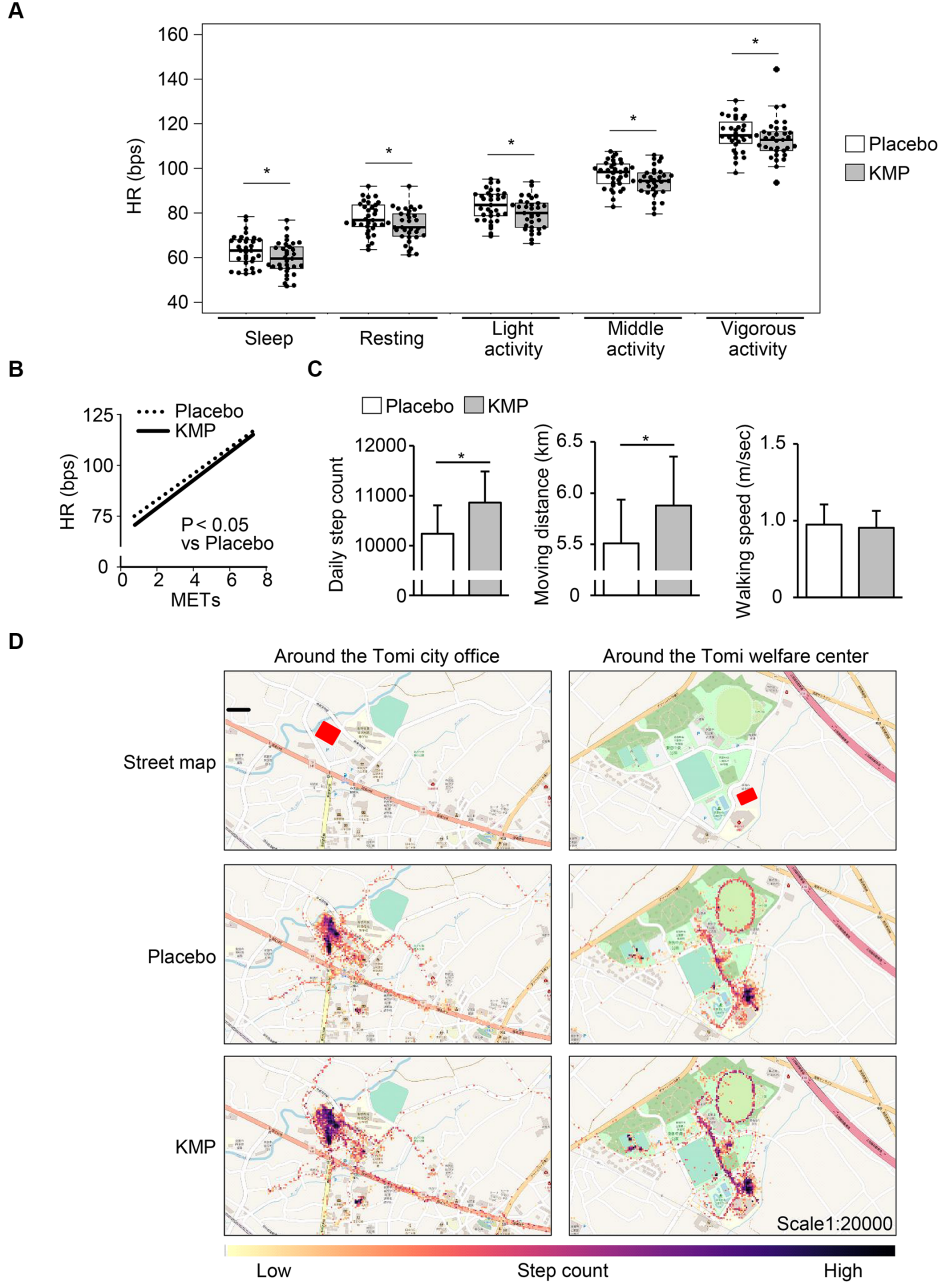
Effects of KMP intake on HR and activity levels. **(A)** HR at each activity level. Data are presented as box plots (median ± interquartile range), and dots represent the data for each participant. *p < 0.05 KMP vs. placebo, mixed model for repeated measures for crossover design. **(B)** Approximate curves obtained by plotting the heart rate during activity for each metabolic equivalent of task (METs) during each supplement intake. Statistical analyses were performed using a mixed model for repeated measures for cross-over design. **(C)** Daily step count, moving distance and walking speed. Data are presented as the mean in the box, and standard error is represented by the error bar for each individual. *p < 0.05 KMP vs. placebo, mixed model for repeated measures for crossover design. **(D)** Heat map created by the number of steps taken per minute.

The intake of KMP-containing supplements decreased HR during light, middle, and vigorous activities by 4 bpm, 3 bpm, and 2 bpm, respectively. Compared with the placebo, KMP intake decreased the HR during sleep and at rest by 6 bpm each (Figure 3A). The physical load index represented the approximated curve obtained by plotting HR on the vertical axis and the exercise intensity (METs) on the horizontal axis, significantly decreased with the intake of KMP-containing supplement (Figure 3B).

The intake of KMP-containing supplements significantly increased the step count and moving distance by 624 steps/day and 0.4 km/day, respectively, compared to the placebo, with no change in walking speed (Figure 3C). The movement of participant in the area around their office increased (Figure 3D).

### 3.3 KMP ingestion led to an increase in weekend activities and recreation

The intake of KMP-containing supplements significantly increased the outing rate and the number of trips on weekends from 91.7 ± 2.6% to 97.7 ± 1.3% and from 3.7 ± 0.2 trips/day to 4.1 ± 0.2 trips/day, respectively (Table 2). Additionally, the intake of KMP significantly increased the number of weekend recreational activities from 0.37 ± 0.05 times/day to 0.53 ± 0.06 times/day, and the weekend time spent on recreation from 1.5 ± 0.3 h/day to 2.2 ± 0.3 h/day, respectively.

**Table 2:** Information on weekend outings and recreation.

	Placebo	KMP
Outing rate on weekends (%)	91.7 ± 2.6	97.7 ± 1.3^*^
Number of trips on weekends (trips/day)	3.7 ± 0.2	4.1 ± 0.2^*^
Number of recreations on weekends (times/day)	0.37 ± 0.05	0.53 ± 0.06^*^
Time spent for recreations on weekends (h/day)	1.5 ± 0.2	2.2 ± 0.3^*^

Data are presented as the mean ± standard error of the mean. ^*^p < 0.05 vs. placebo, mixed model for repeated measures for cross-over design.

### 3.4 KMP ingestion improved sleep quality without affecting sleep time and interruption

**Figure 4:**
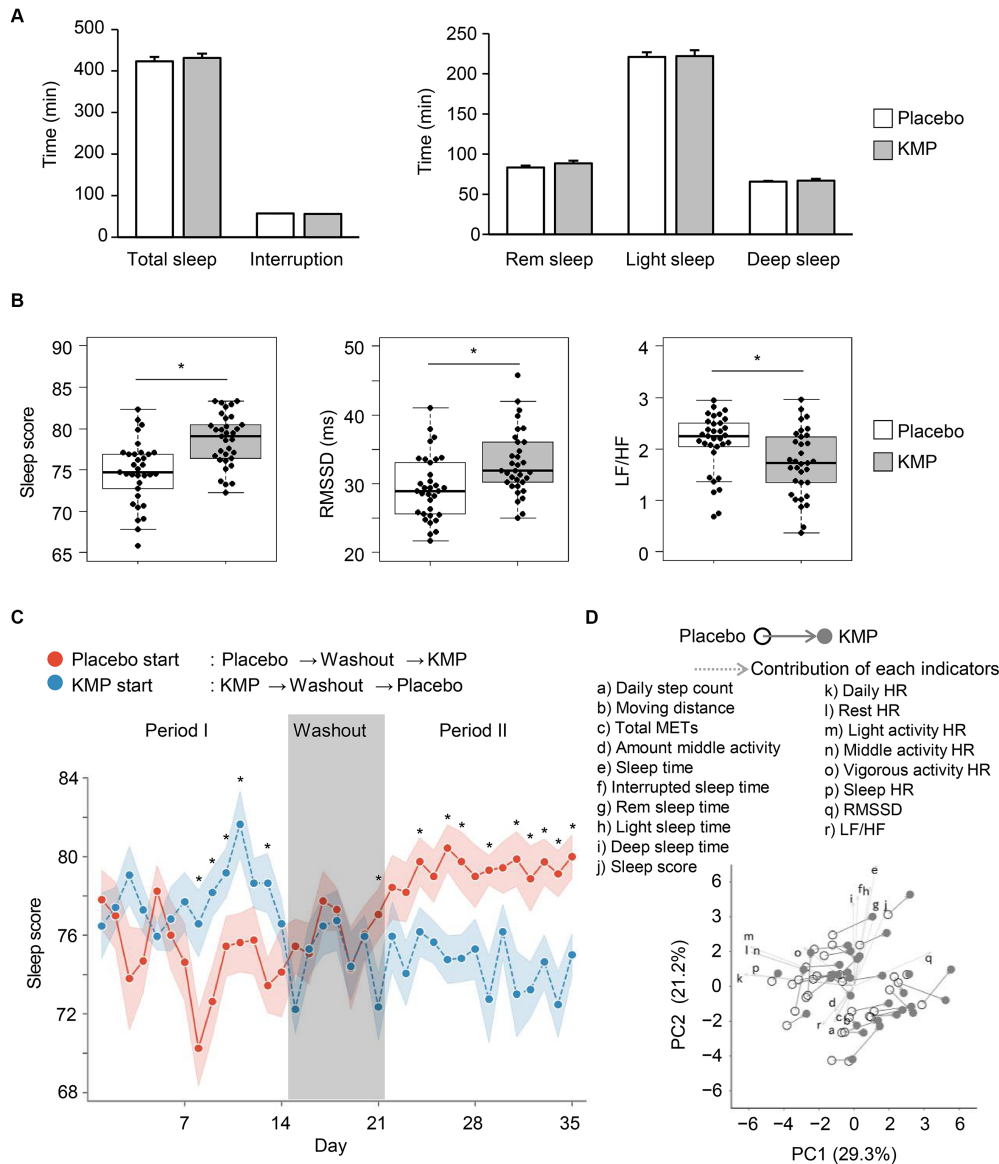
Effects of KMP intake on sleep and regulation of autonomic nervous system. **(A)** Mean time of each sleep stage. Error bars indicate standard error. **(B)** Sleep-related indicators. Data are presented as box plots (median ± interquartile range), and dots represent the data for each participant. *p < 0.05 KMP vs. placebo, mixed model for repeated measures for crossover design. **(C)** Transition of sleep scores throughout the trial period. Circle indicates the mean value, and ribbon represents the range of standard error. *p < 0.05 placebo start group vs. KMP start group, *t*-test. **(D)** PCA using the dataset of the placebo group and KMP group of each participant. Solid arrow indicates a change from placebo to KMP, and dotted arrows represent the contribution of each indicator.

No significant changes were observed in sleep time, sleep interruption time, REM sleep time, light sleep time, and deep sleep time with the intake of KMP-containing supplements (Figure 4A). On the other hand, sleep score and RMSSD significantly increased by 3.9 points and 3.6 ms, respectively, compared to the placebo (Figure 4B). Furthermore, after the intake of KMP-containing supplement, the LF/HF ratio significantly decreased by 0.40 compared to the placebo.

When comparing the trends in sleep scores during the trial period, a significant difference was observed between the placebo and KMP periods, regardless of the test food initiated in the trial. Interestingly, this significant difference disappeared during the washout period (Figure 4C).

In PCA, patterns of change from placebo to KMP were observed across participants with different backgrounds (Figure 4D). While the magnitude of the changes did not significantly surpass the individual differences at baseline, the direction of change was similar. When comparing the direction of change in each participant and the direction of the contribution of each indicator, RMSSD was the most similar index (Supplementary Figure S1).

### 3.5 KMP ingestion improved sleep quality without affecting sleep time and interruption

**Figure 5:**
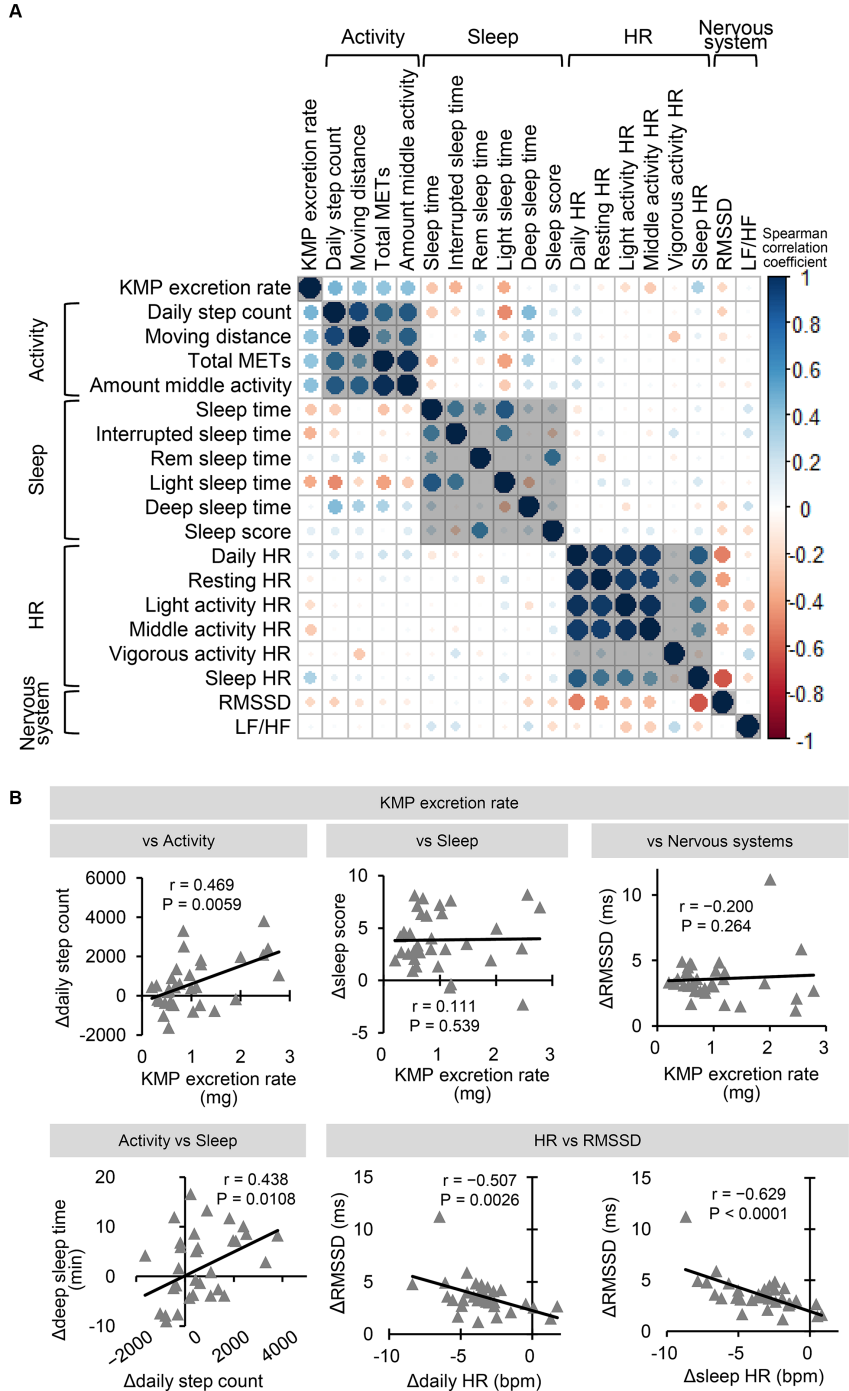
Correlation between changing indicators due to KMP intake. **(A)** Correlation of change amount of each indicator (Δ = KMP – Placebo). The color and size of the circle represent the value of the Spearman correlation coefficient. **(B)** Correlation relationship between change in the levels of the two indicators. Solid line represents the approximation curve.

Figure 5A shows the correlation between the amount of changes of each indicator due to KMP intake. The KMP excretion rate showed a positive correlation with Δdaily step count (Figure 5B), Δmoving distance (*r* = 0.407, *p* = 0.0187), Δtotal METs (*r* = 0.401, *p* = 0.0208) and Δmiddle activity (*r* = 0.416, *p* = 0.0161), and a weak negative correlation with Δlight sleep time (*r* = −0.382, *p* = 0.0282); no correlation with Δsleep score (Figure 5B) and ΔRMSDD (Figure 5B) was found. Δdaily step count correlated positively with Δdeep sleep time (Figure 5B), and Δdaily HR (Figure 5B), Δsleep HR (Figure 5B), and Δresting HR (*r* = −0.417, *p* = 0.0158) correlated negatively with ΔRMSSD.

Supplementary Figure S2A shows the correlation between indices combining data from the placebo and KMP groups. The KMP excretion rate showed a positive correlation with sleep score (Supplementary Figure S2B) and a weak correlation with RMSSD (*r* = 0.382, *p* = 0.0015) and LF/HF (*r* = −0.357, *p* = 0.0033). Sleep HR, daily HR, and LF/HF negatively correlated with RMSSD (Supplementary Figure S2B). Light activity HR (*r* = −0.434, *p* = 0.0003), middle activity HR (*r* = −0.403, *p* = 0.0008), and resting HR (*r* = −0.398, *p* = 0.0009) showed significant or weak negative correlation with RMSSD.

## 4 Discussion

Daily intake of KMP improved both PA and sleep quality. Regular PA is associated with a reduction in mortality and the risk of chronic diseases (36–38). PCA and correlational analysis suggested that the effects of KMP were based on a decrease in HR and an increase in RMSSD. The increase in PA levels depends on KMP absorbability, suggesting that increased blood concentration is directly important. By contrast, sleep quality does not depend on the absorbability of KMP, suggesting that it is effective at low blood concentrations or operates through an indirect mechanism. KMP, a single ingredient, has multiple health benefits through multiple mechanisms, which is intriguing and strongly indicates its usefulness. Our results suggest that KMP, by mediating effects on such as oxygen supply and energy production and improving overall quality of life rather than treating specific diseases, has the potential to offer broad value to individuals and society.

### 4.1 KMP ingestion led to a decrease in HR and an increase in PA

By incorporating KMP intake into daily life without restricting everyday activities, we observed a decrease in HR across all PA levels and an increase in step counts and visual behavioral volumes as shown by the heatmaps. These results are similar to previous survey findings that daily PA increases email and web-based interventions (39–41), suggesting the potential of daily KMP intake as a new tool for enhancing daily PA. Recreation serves as a tool for diversion and stress relief, as well as promoting health (42). Additionally, it contributes to mental well-being.

Even light exercises such as walking or bicycling, when continued, prevent diseases and reduce all-cause mortality rates (43, 44). There is a difference of approximately 1,000 steps per day between individuals with and without fatigue (31). Our previous study has revealed that KMP intake improves VO_2max_, muscle strength, and sleep quality, all of which have a negative correlation with fatigue (45–47). Therefore, the increase in PA observed in this study is likely due to the fatigue-reducing effect of KMP. If this hypothesis is correct, it is noteworthy that HR decreased in all aspects of daily life. HR increases with the accumulation of fatigue and reflects the physical load of daily activities (29), but it has been difficult to evaluate HR and PA levels simultaneously. In this regard, the physical load index evaluation conducted in this study holds potential as a new index for conveniently measuring daily fatigue and the physical load of daily activities.

### 4.2 KMP intake led to sleep quality improvements

HR variability affects PA levels and sleep quality. For example, a lower HR during sleep is associated with improved autonomic function and better sleep quality (48). In fact, the LF/HF ratio decreases with the depth of sleep (49), and lower RMSSD is associated with difficulty falling asleep and frequent awakenings during the night (50). Our findings revealed that KMP intake reduced HR and LF/HF ratio and increased RMSSD during sleep, thereby increasing sleep score, suggesting the potential for KMP intake to provide healthy sleep. On the other hand, self-evaluation of sleep may be more important than objective evaluation of the relationship (51, 52), and we are currently evaluating the relationship between the results of the 3DSS (three-dimensional sleep scale), which can calculate subjective sleep quality, and Fitbit Charge 4 in a separate clinical trial.

There are several potential mechanisms that could lead to improved sleep quality. Dworak et al. reported a positive correlation between deep sleep and brain ATP content (53). In our previous animal studies, in the brains of rats exposed to a low-oxygen environment (12% oxygen), ATP content decreases, but this decrease is mitigated by oral administration of KMP (27). Additionally, a meta-analysis has shown the impact of increased PA on sleep quality (54). Taking these pieces of information together, it can be hypothesized that the improvement in sleep indices due to KMP intake could be due to increased PA, potentially forming a positive feedback loop between oxygen supply, decreasing HR, promoting PA level, regulating the autonomic nervous system, and improving sleep quality.

PCA showed that KMP intake exerts similar effects in participants with variable backgrounds, reinforcing the potential for KMP’s effects to be generalized and widely applied. The changes from placebo to KMP were characterized by RMSSD. However, as mentioned below, the action of KMP on RMSSD may be indirect owing to the increase in PA. Therefore, the effects of food ingredients such as KMP, unlike those of highly selective drugs, are non-selective actions that broadly affect our bodies, yielding various health benefits.

### 4.3 Mechanisms underlying multiple effects of KMP

We conducted a correlation analysis between the change in indicators due to KMP intake to understand the overall effects of KMP and predict its mechanism. The ΔPA level correlated with the absorbability of KMP, suggesting that the absorbability of KMP is likely to be directly involved in the mechanism that improved the PA levels. Therefore, enhancing the bioavailability of KMP could lead to an increase in daily PA levels.

While the sleep score and RMSSD in the KMP group were significantly higher than those in the placebo group, there was no correlation between Δsleep score and ΔRMSSD and absorbability of KMP, suggesting that the effects of KMP on sleep score and RMSSD could be exerted “regardless of the level of absorbability,” or through a “pathway driven by low blood concentration,” or “indirectly such as through metabolically converted components,” or through a “change in PA levels.” The Δdaily step count correlated positively with Δdeep-sleep time. KMP intake did not affect the average sleep time of the whole group but affected the deep-sleep time in individuals who showed changes in PA levels, i.e., those with high absorbability of KMP. These factors show a complex relationship and are presumed to affect sleep quality. Interestingly, ΔRMSSD, which characterizes the change from placebo to KMP, showed a sufficient correlation with ΔHR, suggesting the importance of the relationship between these two indicators. In the correlational analysis combining data from the placebo and KMP groups, a strong inverse correlation was found between RMSSD and HR, suggesting that the decrease in HR and increase in RMSSD are core factors underlying the effects of KMP.

### 4.4 Potential for KMP to solve health problems caused by hypoxia

Hypoxia is a lethal risk to the survival of living organisms and causes energy deficiency. To address hypoxia, the body increases respiratory rate and HR, along with pulmonary artery constriction and HIF activation (55). Among high-altitude residents, some develop chronic mountain sickness or pulmonary hypertension (56, 57) but most are known to adapt to hypoxic environments (58). Their daily diet may underlie adaptation mechanisms.

Environmental stresses, including ultraviolet exposure, increase the content of KMP in plants (59). Plants at high altitudes contain more KMP than those at low altitudes (60), suggesting that the daily intake of KMP-containing plants by high-altitude residents may contribute to their adaptation to the hypoxic environment. Our basic experiments revealed that KMP suppresses HIF-1α stabilization under hypoxia conditions and increases oxygen utilization efficiency in cells with reduced oxygen supply (27).

Flavonoids, including KMPs, generally found in plants, exist as glycosides, and they might not be absorbed even when ingested. The test food we used in this study is easily absorbable, having converted KMP glycoside into aglycone using a unique method. A positive correlation was observed between ΔPA levels and the absorbability of KMP, indicating that providing food containing easily absorbable KMP is important for increasing PA levels and, ultimately, promoting health.

Nagano Prefecture, the site of our study, has a large population of long-lived individuals and an average altitude of over 1,000 m. During the placebo intake period, participants’ mean number of steps per day exceeded 10,000. These participants may contribute to their daily PA and enhance oxygen utilization efficiency by regularly consuming vegetables cultivated at high altitudes.

### 4.5 Limitations and future directions

This study has some limitations that must be addressed. First, the city workers who participated in this study were a group without exercise habits but achieved an average of 10,000 steps per day. The effects of KMP in people with average or below-average exercise habits need further investigation to confirm these results in individuals with low PA levels. Second, this study investigated whether PA changes occurred only when KMP was ingested, without limiting daily activities. Although PA levels increased, future studies need to confirm that this was not correlated with the ingestion of other supplements that may increase PA. Third, subjective assessments may be more important than objective assessments for sleep (48, 49). Polysomnography is commonly used to simultaneously measure electromyography and electro-oculography results (61), and there may have been limitations in these measurements using a simple wearable device. Future studies need to investigate whether KMP ingestion improves objective and subjective sleep indices while elucidating the underlying mechanism of action. Finally, there was a significant correlation between HR and RMSSD, and is a core relationship mediating the effect of KMP. KMP may exert its effects through a direct mechanism in PA or secondary metabolites or indirect mechanisms involving sleep quality. Therefore, while increasing the absorption of KMP is crucial for enhancing PA levels, the improvement in sleep quality is not related to the amount of KMP absorbed, suggesting that it can benefit everyone. At this point, we interpret that the improvement in sleep quality was owing to the increase in PA. In the future, clarifying the relationship between KMP intake and sleep quality will be necessary to better understand the phenomena occurring throughout the body.

There is a positive correlation between VO_2max_ and frontal lobe function (57), as well as mental health that controls emotions (41). Our findings suggested that HR and RMSSD values are involved. Therefore, fluctuations in the oxygen supply-energy supply-HR axis may be important for performance during activity and regulating the autonomic nervous system at rest. KMP affected these control systems but the causal relationship and molecular mechanism were not examined and need to be clarified in future studies.

## 5 Conclusion

The daily intake of KMP leads to behavioral changes, including an increase in PA and the number of outings on weekends, which subsequently improve sleep quality. Furthermore, we have elucidated the relationship between PA and sleep. The associated decrease in HR and regulation of the autonomic nervous system may have played important roles in these changes. Generally, the effects of food are not as potent as those of pharmaceuticals; however, KMP could potentially be used as a food ingredient that can help maintain the quality of life for people living in the era of a 100-year lifespan, considering that it can be ingested daily. Future research should focus on elucidating the detailed molecular mechanisms of KMP, and its potential contribution to the lives of people with diverse backgrounds.

## Data availability statement

The raw data supporting the conclusions of this article will be made available by the authors, without undue reservation.

## Ethics statement

The studies involving humans were approved by Ethics Committee of Otsuka Pharmaceutical Co., Ltd. (approval no. 2003, dated October 30, 2020). The studies were conducted in accordance with the local legislation and institutional requirements. The participants provided their written informed consent to participate in this study.

## Author contributions

YI: Conceptualization, Data curation, Formal analysis, Investigation, Methodology, Project administration, Supervision, Visualization, Writing – original draft, Writing – review & editing. AG-K: Formal analysis, Visualization, Writing – review & editing. SO: Investigation, Methodology, Validation, Writing – review & editing. SH: Investigation, Methodology, Validation, Writing – review & editing. TS: Investigation, Methodology, Validation, Writing – review & editing. TM: Data curation, Investigation, Methodology, Validation, Writing – review & editing. BS: Supervision, Writing – review & editing.

## Supplementary material

Supplementary Figure S1 Contribution degree of each indicator in the changes owing to KMP intake. The similarity of the vector direction from placebo to KMP with each indicator’s vector was calculated. Cosine similarity is shown in the box plots (median ± interquartile range), and dots represent the data for each participant.

Supplementary Figure S2 Correlation relationship between each indicator. **(A)** Correlation of each indicator throughout placebo and KMP periods. The color and size of the circle represent the value of the Spearman correlation coefficient. **(B)** Correlation relationship between two indicators. Solid line represents the approximation curve.
