# Supplementary figures and images for: Effect of kaempferol ingestion on physical activity and sleep quality: a double-blind, placebo-controlled, randomized, crossover trial
Source: Front Nutr. 2024 Aug 2;11:1386389. doi: 10.3389/fnut.2024.1386389 (PMC11327823; doi:10.3389/fnut.2024.1386389)

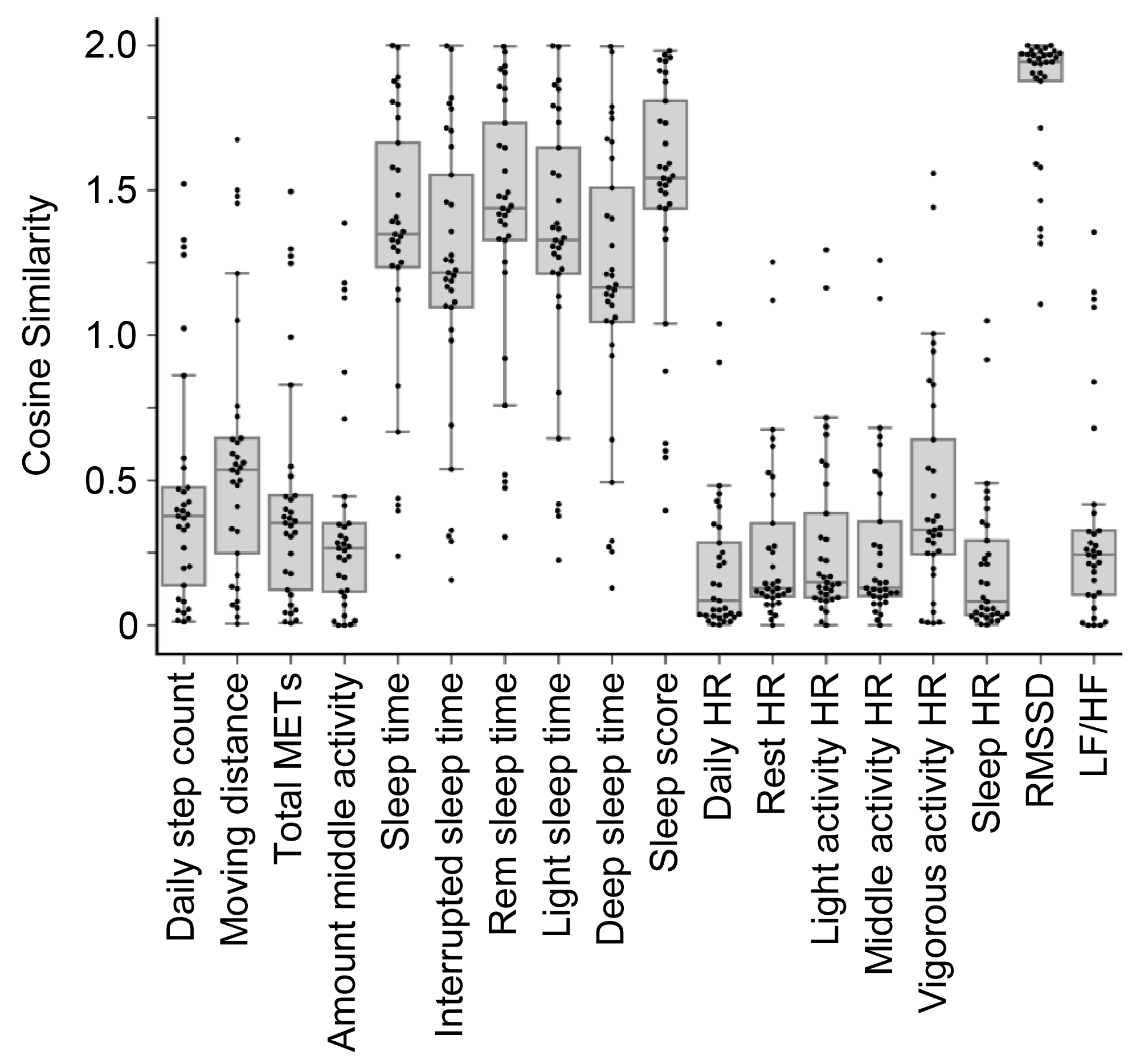

Supplement: Supplementary Figure S1 — Contribution degree of each indicator in the changes owing to KMP intake. The similarity of the vector direction from placebo to KMP with each indicator’s vector was calculated. Cosine similarity is shown in the box plots (median ± interquartile range), and dots represent the data for each participant. [file Image_1.TIF]

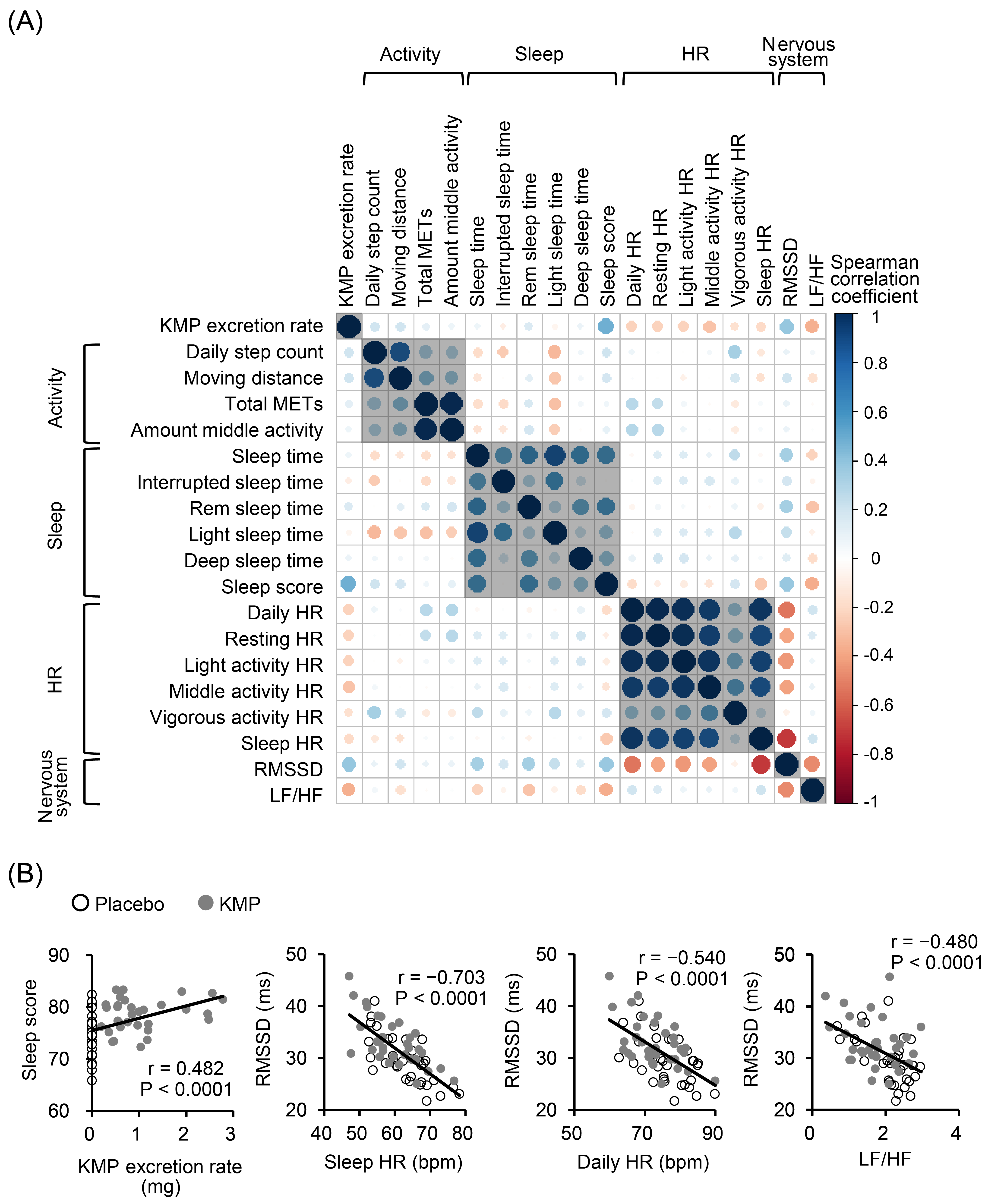

Supplement: Supplementary Figure S2 — Correlation relationship between each indicator. (A) Correlation of each indicator throughout placebo and KMP periods. The color and size of the circle represent the value of the Spearman correlation coefficient. (B) Correlation relationship between two indicators. Solid line represents the approximation curve. [file Image_2.TIF]
